# In vitro and in vivo evaluation of a tetrazine-conjugated poly-L-lysine effector molecule labeled with astatine-211

**DOI:** 10.1186/s41181-024-00273-z

**Published:** 2024-05-22

**Authors:** Chiara Timperanza, Holger Jensen, Ellinor Hansson, Tom Bäck, Sture Lindegren, Emma Aneheim

**Affiliations:** 1 Department of Medical Radiation Sciences, Institute of Clinical Sciences, Sahlgrenska Academy, University of Gothenburg, Gothenburg, 413 45 Sweden; 2 Department of Clinical Physiology and Nuclear Medicine, Cyclotron and Radiochemistry unit, Rigshospitalet, Blegdamsvej 9, Copenhagen, 2100 Denmark; 3 Department of Oncology, Sahlgrenska University Hospital, Region Västra Götaland, Gothenburg, 413 45 Sweden; 4 Atley Solutions AB, Gothenburg, 413 27 Sweden

**Keywords:** Targeted therapy, Targeted alpha therapy, Astatine-211, Pretargeted radioimmunotherapy, Click chemistry, Tetrazine-TCO

## Abstract

**Background:**

A significant challenge in cancer therapy lies in eradicating hidden disseminated tumor cells. Within Nuclear Medicine, Targeted Alpha Therapy is a promising approach for cancer treatment tackling disseminated cancer. As tumor size decreases, alpha-particles gain prominence due to their high Linear Energy Transfer (LET) and short path length. Among alpha-particle emitters, ^211^At stands out with its 7.2 hour half-life and 100% alpha emission decay. However, optimizing the pharmacokinetics of radiopharmaceuticals with short lived radionuclides such as ^211^At is pivotal, and in this regard, pretargeting is a valuable tool. This method involves priming the tumor with a modified monoclonal antibody capable of binding both the tumor antigen and the radiolabeled carrier, termed the “effector molecule. This smaller, faster-clearing molecule improves efficacy. Utilizing the Diels Alder click reaction between Tetrazine (Tz) and Trans-cyclooctene (TCO), the Tz-substituted effector molecule combines seamlessly with the TCO-modified antibody. This study aims to evaluate the in vivo biodistribution of two Poly-L-Lysine-based effector molecule sizes (10 and 21 kDa), labelled with ^211^At, and the in vitro binding of the most favorable polymer size, in order to optimize the pretargeted radioimmunotherapy with ^211^At.

**Results:**

In vivo results favor the smaller polymer’s biodistribution pattern over the larger one, which accumulates in organs like the liver and spleen. This is especially evident when comparing the biodistribution of the smaller polymer to a directly labelled monoclonal antibody. The smaller variant also shows rapid and efficient binding to SKOV-3 cells preloaded with TCO-modified Trastuzumab in vitro, emphasizing its potential. Both polymer sizes showed equal or better in vivo stability of the astatine-carbon bond compared to a monoclonal antibody labelled with the same prosthetic group.

**Conclusions:**

Overall, the small Poly-L-Lysine-based effector molecule (10 kDa) holds the most promise for future research, exhibiting significantly lower uptake in the kidneys and spleen compared to the larger effector (21 kDa) while maintaining an in vivo stability of the astatine-carbon bond comparable to or better than intact antibodies. A proof of concept in vitro cell study demonstrates rapid reaction between the small astatinated effector and a TCO-labelled antibody, indicating the potential of this novel Poly-L-Lysine-based pretargeting system for further investigation in an in vivo tumor model.

**Supplementary Information:**

The online version contains supplementary material available at 10.1186/s41181-024-00273-z.

## Background

Cancer cells that escape their primary tumor, survive in the circulation, and attach to distant organ are called disseminated tumor cells (Chambers et al. 2002). Eradicating these types of cancer cells is crucial to improve treatment outcomes (Pantel and Fodstad 1999). For the treatment of such metastasis, current strategies include chemotherapy, immunotherapy, and targeted therapy, sometimes used in combination (Ganesh and Massague 2021). Targeted Alpha Therapy (TAT) using ^211^At is an emerging and promising approach (Zalutsky et al. 2007; Guerra Liberal et al. 2020) which falls under the broader category of targeted radionuclide therapies, where a radioactive isotope attached to a targeting vector is used to selectively target and destroy cancer cells while minimizing damage to healthy tissue (Makvandi et al. 2018; Working 2018). Alpha particles in particular are highly energetic heavy particles with a short range in tissue, which means that they can deposit a significant amount of energy in a small area. This makes them highly effective at killing isolated cancer cells or cell clusters while sparing the surrounding healthy tissue (Working 2018), reducing the side effects compared to other radiation therapies such as those employing different types of radiation qualities like beta-minus emitters. These particles possess a lower linear energy transfer (LET) and a broader tissue range compared to alpha particles, and are therefore more suitable for the treatment of larger tumors (Working 2018; Eychenne et al. 2021; Zalutsky 1996). ^211^At is particularly attractive due to its unique properties as an alpha-emitting radionuclide. The relatively short half-life (7.21 h) enables both, time for production and in vivo distribution of the radiopharmaceutical, and at the same time, allows for faster clearance from the body (Guerard et al. 2013). Additionally, the simple decay scheme of ^211^At presents a significant advantage compared to other alpha emitters (e.g., ^225^Ac) avoiding potentially toxic daughters which complicate dosimetry (Working 2018; Eychenne et al. 2021). To deliver ^211^At to the tumor, it is typically attached to a (bio)molecule that can specifically target cancer cells. In general treatments the radiopharmaceutical is administered systemically to reach dispersed tumor cells. However, in this case the half-life of ^211^At would not allow for a sufficient absorbed dose to the tumor unless a fairly small molecule with fast distribution is used as a vector (Zalutsky et al. 2007). For this reason, pretargeted radioimmunotherapy (PRIT) could be a favorable strategy to optimize the pharmacokinetics of ^211^At-labeled radiopharmaceuticals (Altai et al. 2017; Cheal et al. 2022). PRIT aims to reduce the overall radiation dose associated with directly labeled antibodies used in radioimmunotherapy (RIT), a form of targeted therapy, improving the therapeutic index (TI) or tumor-to-normal-tissue absorbed dose ratio (Cheal et al. 2022; Jallinoja and Houghton 2021). A common approach is the use of a modified monoclonal antibody (mAb) as pretargeting agent, recognizing the tumor antigen on cancer cells. In pretargeting such an antibody is modified for binding to a small molecule carrying the radionuclide, the effector molecule (Verhoeven et al. 2019). The effector molecule should be sufficiently small to readily reach the tumor and be designed not to accumulate in radiosensitive organs, such as the kidneys. However, excessively small effector molecules may require an addition of a carrier (e.g., albumin 66.5 kDa) to prolong their circulation and to prevent their rapid excretion. Thus, the size of the effector molecule plays a crucial role in striking the appropriate balance between tumor uptake and blood/body clearance of the activity. Utilizing a polymer scaffold ranging from 10 to 21 kDa as effector molecule enables the attachment of a greater number of prosthetic groups for the radionuclide and pretargeting functional groups per effector compared to small molecules. This enhances the avidity to the PRIT-agent, thereby improving its effectiveness. The biorthogonal click reaction between Tetrazine (Tz) and Trans-cyclooctene (TCO) is a key component of several studied PRIT strategies (Bauer et al. 2023; Handula et al. 2021; Png et al. 2017). Being bioorthogonal means that it occurs selectively and rapidly in biological systems without interfering with other cellular processes, giving high yields and minimal side reactions (Bird et al. 2021). These features allow for the precise and efficient attachment of targeting moieties to the tumor-targeting molecule, resulting in enhanced tumor localization and therapeutic efficacy while minimizing off-target effects. In our previous work, our primary objective was to create and assess a novel category of effector molecules for pretargeting applications involving ^211^At. This was accomplished using a poly-L-lysine (PL) scaffold and capitalizing on the Tz–TCO click reaction. We explored various chemical aspects, including the influence of the polymer chain length and functionalization, the selection of the radionuclide and the in vitro stability of these effector molecules (Timperanza et al. 2023). The aim of our current study was to evaluate in vivo biodistribution of astatine labelled PL effectors of different sizes and to make a first assessment of the in vitro binding characteristics when combined with a TCO-modified antibody, which serves as the pretargeting agent.

## Results

Two different PL molecules of different molecular weights (large 21,000 g/mol and small 10,000 g/mol) were successfully conjugated for astatination and click-reaction with TCO according to a previously reported protocol where all details can be found (Timperanza et al. 2023). In brief, the large polymer conjugation results in approximately 13 lysines being functionalized with tetrazines and approximately 13 lysines with the prosthetic groups for attaching the radionuclide. Conjugation of the small polymer yields approximately 3 tetrazines and 3 prosthetic groups. The residual lysines are functionalized with succinic anhydride for charge modification of the polymer. This is necessary to avoid losses as the otherwise highly positively charged polymer sticks to plastic and glass surfaces of vials, columns et.c. Unfortunately, the extent of succinylation cannot be assessed due to the structure of the polymer but the successful recovery of the polymer after the above-mentioned modifications indicate that it is efficient. The high reproducibility of this process can be reflected in the very consistent results of the subsequent radiolabeling with ^211^At of the two effector molecules. In the current study, the radiochemical yield (RCY) was 76.16% for the large PL effector and 84.87% for the small PL effector, which can be compared to previously reported numbers of 85.38% ± 0.02 and 84.78% ± 0.03 respectively (*n* > 20) (Timperanza et al. 2023).The radiochemical purity (RCP) was > 97% for both sizes. The specific molar activity for the large and the small PL in the current animal study was 18 and 9 MBq/nmol respectively.

### Stability in serum

In a previous study, the labeled polymers showed favorable in vitro radio-stability over 24 h in PBS as determined with fast protein liquid chromatography (FPLC) and radio-TLC (Timperanza et al. 2023). In the current study the in vitro stability of both sizes (10 kDa and 21 kDa) of the astatinated effector molecules and the clicked products, the latter obtained from the in vitro reaction between the labeled TzPLs and TCO-Trastuzumab, was investigated in the presence of human serum albumin (HSA). After incubating for 24 h at 37 °C, FPLC analyses revealed that both effectors and clicked products are stable in the presence of HSA in a concentration similar to that in blood, mimicking blood serum, reporting no changes in the chromatograms (supporting information Figure S1).

### In vivo biodistribution of the effectors

**Fig. 1:**
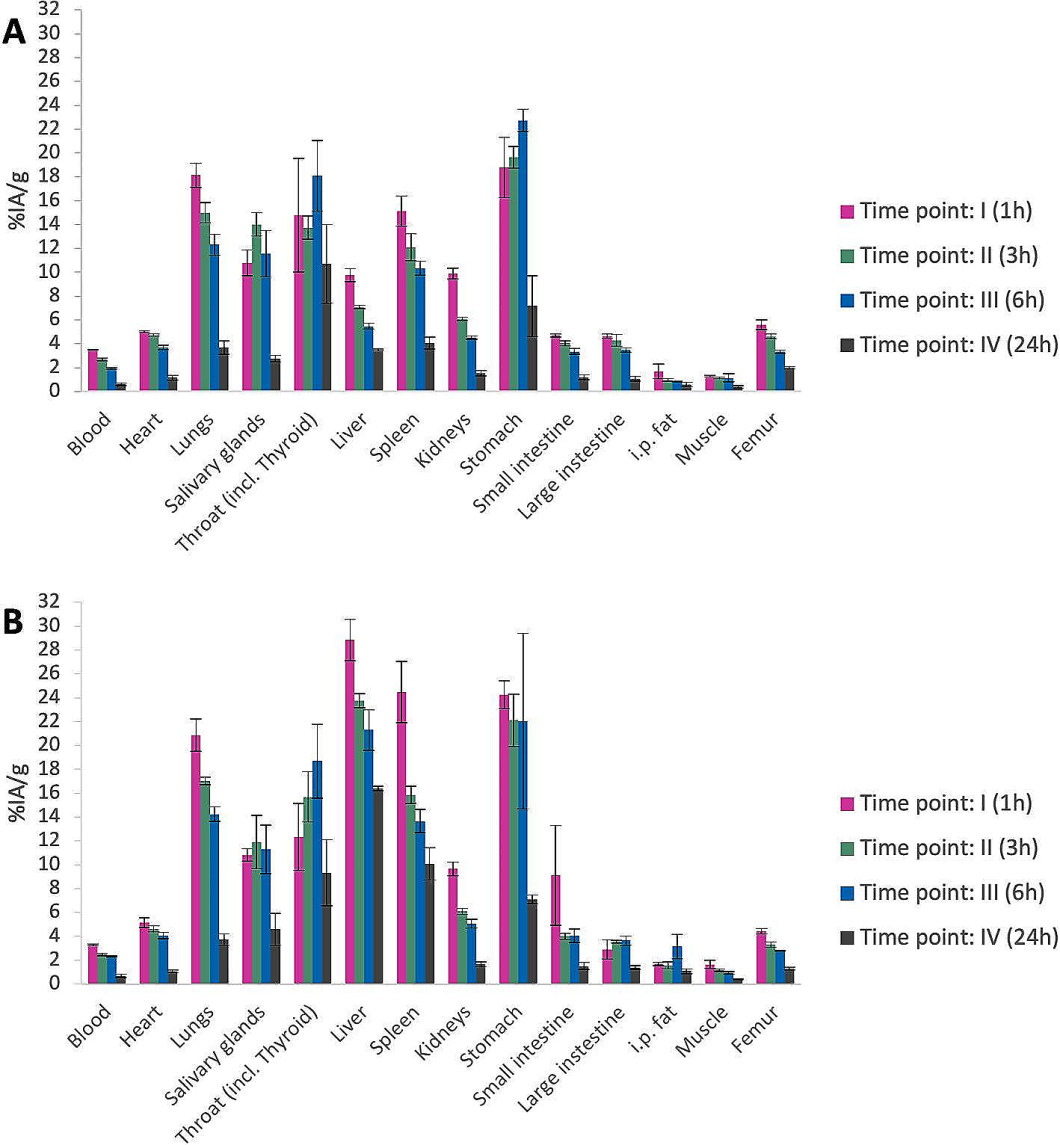
Biodistribution studies in healthy female BALB/c mice of ^211^At-labeled poly-L-lysine-based (^211^At-TzPL) effector molecules of two sizes (**A**) 10 kDa and (**B**) 21 kDa. The results are reported as the percentage of the injected activity per gram of tissue (%IA/g; *n* = 3) at 1, 3, 6, and 24 h after intravenous injection (IV). Error bars are reported as standard error of the mean (SEM)

**Fig. 2:**
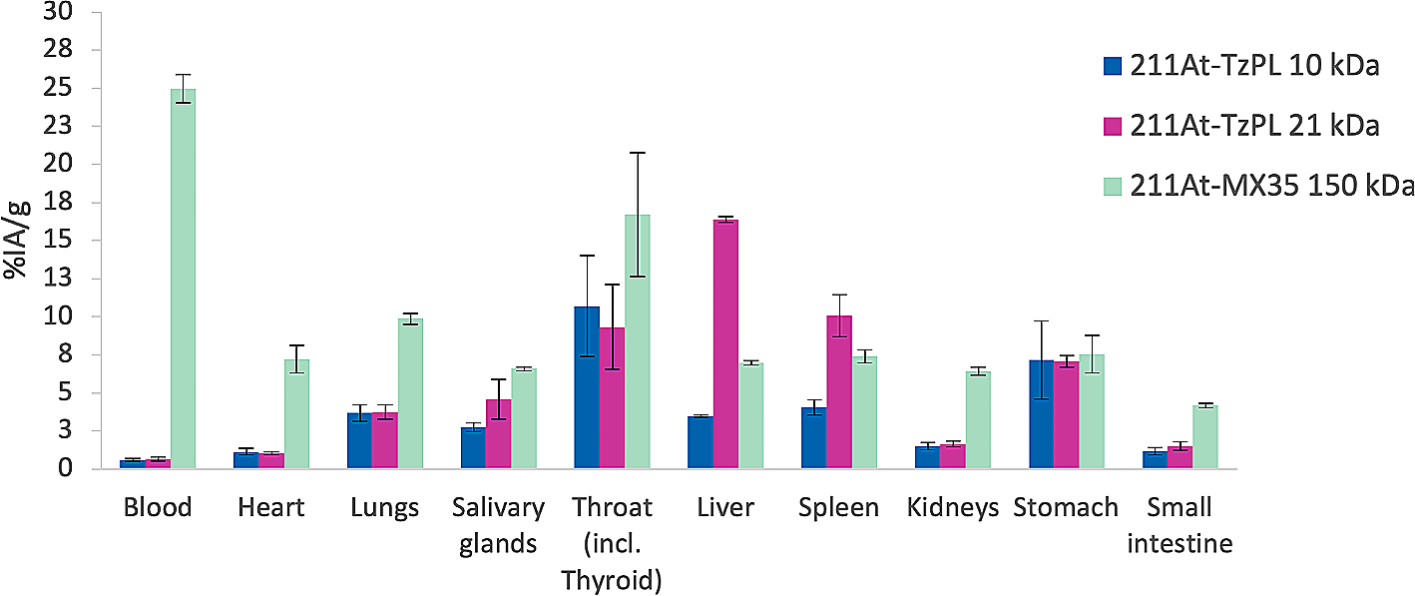
Comparison of the 24 h biodistribution time point of astatinated poly-L-lysine-based (^211^At-TzPL) effector molecules of two sizes (10 kDa and 21 kDa) and an astatinated monoclonal antibody (mAb) MX35 (^211^At-MX35; 150 kDa). Both effector molecules and the mAb were conjugated with the same activated tin ester (ATE) for astatination. The results are reported as the percentage of the injected activity per gram of tissue (%IA/g) 24 h after the injection on healthy female BALB/c mice. Error bars are reported as standard error of the mean (SEM)

In this work, an in vivo biodistribution study of the two different PL effector molecules was conducted in a tumor-free mouse model in order to assess the pharmacokinetics profile, including the organ retention, and the in vivo radio-stability of the astatinated effectors. Results show generally higher organ retention of the large PL effector compared to the small PL effector, Fig. 1. This was particularly evident in the liver and in the spleen, where after 24 h post-injection, the percentage of injected activity per gram was circa four and three times higher for the 21 kDa PL effector compared to the 10 kDa PL effector. It is acknowledged that unbound ^211^At tends to accumulate primarily in the thyroid and stomach, with additional accumulation observed in the lungs, spleen, and salivary glands (Ukon et al. 2020). A higher uptake in thyroid and stomach is commonly observed also when comparing directly astatinated and iodinated antibodies (Lindegren et al. 2008). The retention of activity in these organs demonstrated reduced stability of the astatine-carbon bond in vivo compared to the in vitro findings. The instability observed in vivo is due to the complex way injected molecules interact with proteins and enzymes in different tissues; an environment that cannot be reproduced in the simpler in vitro conditions. Notably, the retention in the ^211^At accumulating organs was nearly identical for both sizes of polymer effectors, suggesting that the length or substitution of the polymer does not impact the in vivo radio-stability of the effector, as depicted in Fig. 1. The biodistribution results at 24 h is also compared with a full-size IgG antibody, MX35, astatinated using the same prosthetic group as the PL effectors. MX35 was chosen as this antibody previously has been used for direct targeting in radioimmunotherapy (RIT) with ^211^At, both in several preclinical studies and also the FAb2 fragment clinically (Aneheim et al. 2016; Cederkrantz et al. 2015; Gustafsson et al. 2012; Andersson et al. 2009). From the results it can be concluded that the activity retention, even for the large PL effector, is considerably shorter in most organs except for the liver and spleen, Fig. 2. In addition, the in vivo stability of the astatine-carbon bond in both PL effectors is equal to or even slightly better than the antibody, as seen by the similar or slightly lower activity uptake in thyroid and stomach.

### Activity blood profile

**Fig. 3:**
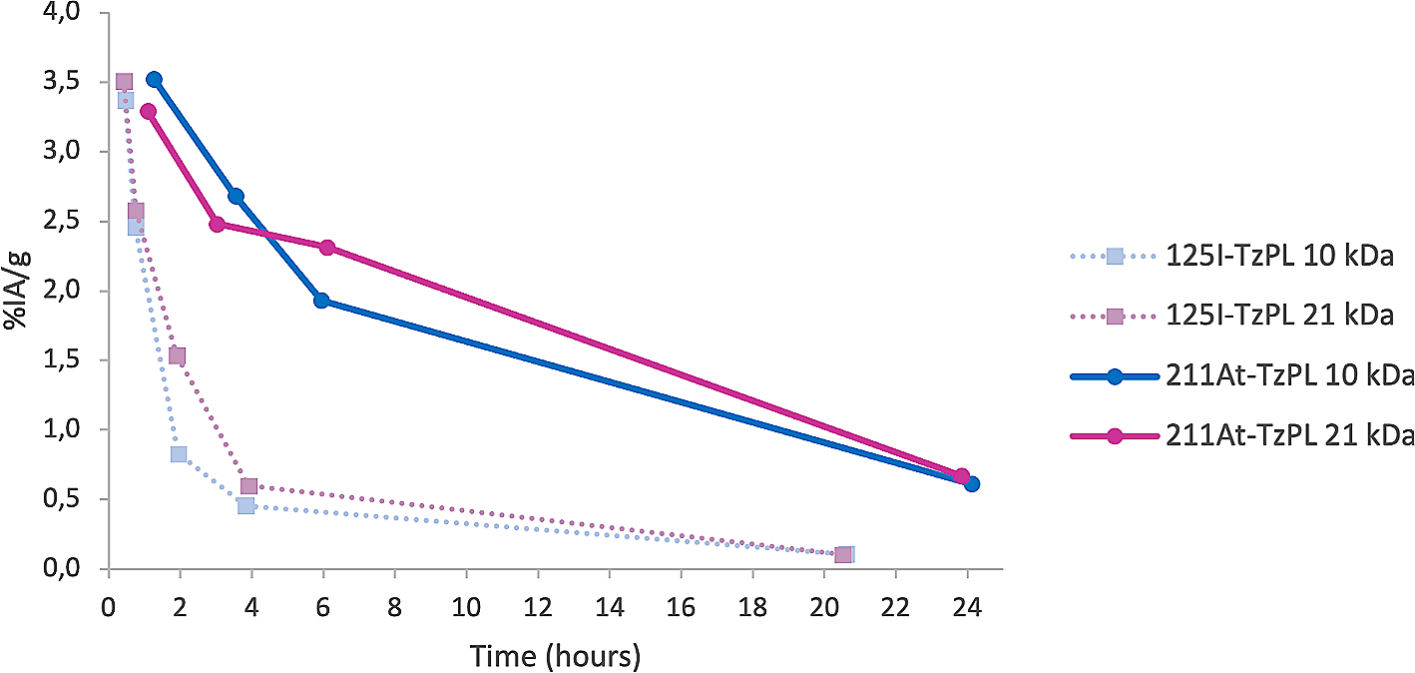
Blood activity profiles reported as the percentage of injected activity per gram (%IA/g) over time in healthy female Balb/C mice of two sizes of astatinated poly-L-lysine (PL) effector molecules (10 kDa and 21 kDa) compared to iodinated PL effectors of the same sizes from a previous study (Timperanza et al. 2023)

The blood samples taken during the biodistribution study show no difference in the blood clearance rate between the two PL effector sizes and the activity is mainly cleared after 24 h, Fig. 3. However, when comparing the blood profile curve of both astatinated PL effectors with the corresponding iodinated versions from a previous study (Timperanza et al. 2023) a clear difference can be seen, showing a higher degree of blood activity retention over time for the astatinated effectors, Fig. 3. The only difference between the two sets of effectors is the radionuclide, as the conjugation of the PL scaffolds is performed in the exact same way.

### Pretargeting cell assay

**Fig. 4:**
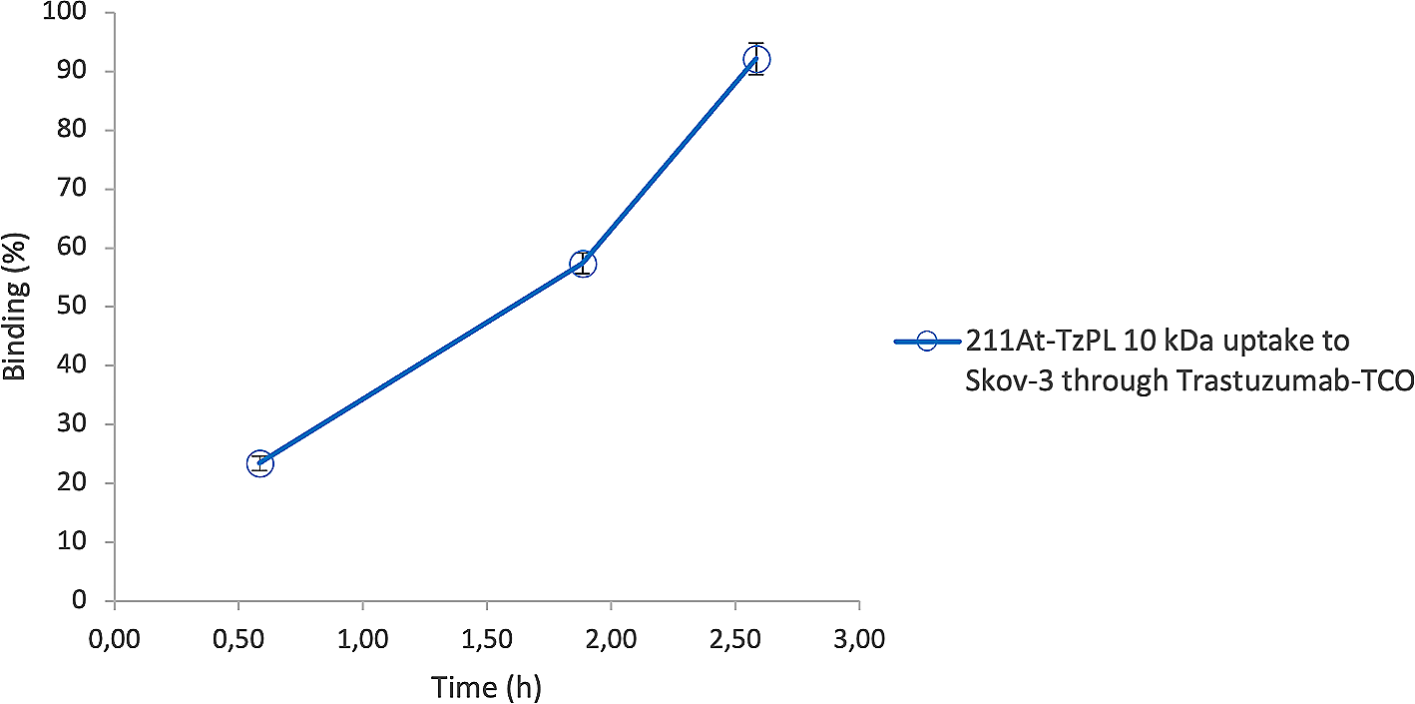
Cell binding study of the small (10 kDa) ^211^At-labeled poly-L-lysine-based (PL) effector molecule to SKOV-3 cells pre-treated with transcyclooctene (TCO)-modified Trastuzumab. Error bars are reported as standard error of the mean (SEM)

As the biodistribution showed favorable results for the smaller PL effector molecule, this was subjected to an in vitro evaluation of the full pretargeting system. The system was tested on a single-cell suspension of SKOV-3 cells utilizing TCO-labelled Trastuzumab as the pretargeting agent to assess click-chemistry kinetics and efficiency of the effector. After uptake of the pretargeting agent on the cells and change of media, the ^211^At-labeled small effector molecule was introduced to the cell suspension in deficit compared to the assumed number of bound pretargeting agents. After incubation for 2.5 h, 92.1% ± 6.8 (mean ± SD; *n* = 4) of the PL effector molecule had bound to the pretargeted SKOV-3 cells, Fig. 4.

## Discussion

This study aimed to evaluate the biodistribution and cell binding of an astatinated poly-L-lysine-based effector molecule of two different sizes. The main goals were to determine which PL size would be more suitable to use for pretargeting applications based on its biodistribution profile and to investigate the in vivo radio stability of the astatine bond in this new type of molecule. This is particularly important due to the known instability of the astatine-carbon bond, which could be affected by the chemical properties of the vector (Zalutsky 2008). As expected, the small PL effector displayed a faster clearance and showed a generally better biodistribution than the large PL effector whereas in vivo deastatination was similar, with a low accumulation of activity in NIS symporter-expressing tissues. Comparisons with a full-size antibody, astatinated using the same prosthetic group as the PL effectors, show similar or slightly better in vivo stability of the formed astatine carbon bond in the PL effector. In pretargeting, the main aim is to use a small, labeled effector molecule so that it can penetrate the tumor environment and at the same time have a fast clearance. In this way the normal tissue distribution and dose to tumor can be optimized (Cheal et al. 2022). From the in vivo study, it was shown that the 21 kDa PL effector not only has a slower clearance but also accumulates in the liver and the spleen. This could be due to the fact that being larger in size, the lipophilicity of the lysine chain increases, leading to increased retention. Moreover, as previously determined by spectrophotometry, the higher molecular weight of the PL effector results in a higher number of tetrazines attached to the PL chain (Timperanza et al. 2023). Due to the aromatic structure of tetrazine the overall lipophilicity of the effector increases further, leading to hepatic clearance (Qiu et al. 2019). In a previous study investigating a PL effector molecule for another PRIT strategy (the streptavidin-biotin system), it was shown that molecular weights lower than 19 kDa ended up in the kidneys (Lindegren et al. 2002), which are one risk organ to consider when determining radiation dose (Bäck et al. 2009). However, in this study, neither of the two PL effector sizes (10 and 21 kDa) showed kidney retention, leading to the conclusion that it is not only the length of the polymer but also the different ligands attached to the PL chain that influence clearance. Finding the right effector size is also important since small organic molecules are generally cleared from the body extremely fast, preventing them from accumulating at the tumor site. Contrary to small molecules being cleared too fast, big molecules can instead be retained in the bloodstream for too long, which is the case with monoclonal antibodies used in radioimmunotherapy (Tabrizi et al. 2010; Ryman and Meibohm 2017; Press et al. 2001). Comparing blood activity for the PL effectors in this study with a full-length monoclonal antibody used in RIT, clearly shows that the PL effectors have blood retention that is better aligned with the half-life of ^211^At. This highlights the benefits of using a PRIT system for systemic treatments involving short-lived radionuclides. The activity blood profile in this work was similar for both PL effector sizes, and the residual activity left in the blood from the astatinated effector molecule was reduced by ∼ 80% at 24 h past injection. Comparison to the corresponding iodinated effector molecules from previous work showed that the astatinated PLs displayed a different blood activity profile, characterized by a slower clearance from the bloodstream. This phenomenon may be attributed to the increased release of free ^211^At from the polymer as compared to iodine due to the weaker carbon-astatine bond in contrast to the carbon-iodine bond (Zalutsky 2008). Additionally, biodistribution studies have demonstrated that free ^211^At exhibits a relatively slow clearance compared to free iodine from the bloodstream and also retention in the stomach, lungs, and spleen (Ukon et al. 2020; Spetz et al. 2013; Larsen et al. 1998). Stability studies were carried out in vitro for the two different sizes of effector molecules and their resulting products formed through a reaction with the TCO-substituted pretargeting agent. These studies, performed in serum at 37 °C for 24 h, demonstrated that the molecules and products did not degrade or undergo significant changes in their structure when subjected to conditions mimicking those found within the human body. Since the small polymer exhibited more favorable biodistribution results, we conducted an in vitro study to assess how quickly the effector molecule binds to a TCO-modified mAb (Trastuzumab) that had been preloaded onto SKOV3 cells. As anticipated due to the rapid reaction kinetics of click chemistry, in particular of the inverse electron demand Diels-Alder system (Sarrett et al. 2021), the effector binding was over 92% within just 2.5 h. This also indicates a low trans-to-cis isomerization of the TCO-conjugated antibody under the present conditions. It is also known that the in vivo half-life of TCO ranges from ∼ 4 to ∼ 10 days depending on the structure of the moiety (Sarrett et al. 2021). This result bodes well for the potential tumor uptake in an in vivo setting which will be evaluated in future studies.

## Conclusions

Taken together, the small PL effector shows the best promise for future studies due to the significantly lower uptake in the kidneys and spleen, with maintained in vivo radio stability, which is equally good or better than for intact antibodies. An in vitro proof of concept cell study shows a fast reaction between the small astatinated PL effector and a TCO-labelled antibody, providing evidence that this PL-based pretargeting system merit further studies in in vivo tumor models.

## Materials and methods

### General

^211^At was obtained from the PET and Cyclotron Unit at Copenhagen University Hospital (Denmark). The nuclide was transformed into a chemically useful form at the Sahlgrenska Academy (Gothenburg, Sweden) by dry distillation using an Atley C100 system, Atley Solutions AB, Gothenburg (Sweden) (Pantel and Fodstad 1999). The N-succinimidyl- 3-trimethylstannyl-benzoate (m-MeATE) bifunctional labeling reagent, 97% purity, was purchased from Toronto Research Chemicals, Inc. Toronto (ON), Canada; 10,000 and 21,000 Da poly-L-lysine hydrobromide were purchased from Alamanda Polymers Inc. Huntsville (AL), USA; TCO-NHS ester (TCO) was purchased from Click Chemistry Tools, Scottsdale (AZ), USA; all other chemicals included in this study were obtained from Sigma Aldrich, Inc. St. Louis (MO), USA, and were of at least analytical grade. The monoclonal antibody (mAb) Trastuzumab (Herceptin®) was obtained from Apoteket AB, Sahlgrenska University Hospital (Gothenburg, Sweden). This mAb is specific for human epidermal growth factor ErbB2 (HER2). The mAb MX35 was developed at the Sloan-Kettering Cancer Center (New York, NY, USA) but was produced from hybridoma cells kindly provided by the Ludwig Institute for Cancer Research, Zürich, Switzerland. This mAb recognizes the sodium- dependent phosphate transport protein 2b (NaPi2b), which is expressed in a number of cancer cell lines. The human cell line SKOV-3, an ovarian carcinoma cell line, was obtained from the American Type Culture Collection (Rockville, Md). The tumor cells were cultured at the Department of Oncology at Sahlgrenska University Hospital (Gothenburg, Sweden).

### Effector molecule synthesis

The polymer was conjugated in advance before radiolabeling according to the protocol reported in our previus study (Timperanza et al. 2023). In brief: two molecular weights, 10 kDa (small PL) and 21 kDa (large PL) were dissolved in 0.2 M carbonate buffer (pH 8.5) at a concentration of 4 mg/mL. From a 50 mg/mL stock solution of m-MeATE in chloroform, an aliquot was transferred to a glass micro vial (1.1 mL V-vial, VWR), and the solvent evaporated. The residue was dissolved in dimethyl sulfoxide (DMSO), resulting in a concentration of 115 nM. The m-MeATE was added to the PL solution and allowed to react at room temperature (RT) under gentle agitation for 30 min. Then, Tetrazine-NHS ester (H-Tz) was dissolved in DMSO to a concentration of 200 mg/mL and added to the reaction mixture, and the reaction proceeded for another 30 min. Solid succinic anhydride was then added in four times molar excess. The pH was adjusted with 20 μL of 1 M sodium carbonate to maintain a level of around 8.5. After 30 min, the resulting conjugated polymer was purified by size exclusion chromatography using a NAP-10 column Cytiva–GE Healthcare, Buckinghamshire (UK). The product was eluted in a PBS solution at pH 7.4.

### Effector molecule radiolabeling

Upon labeling, the buffer of the conjugated polymer was exchanged from PBS to 0.2 M acetate, pH 5.5, to enable the ^211^At-labeling reaction. Astatination was performed starting with 109–111 MBq of ^211^At in chloroform obtained from the irradiated target. The solvent was evaporated under a gentle nitrogen stream to obtain a dry residue of ^211^At, to which N-iodosuccinimide (NIS) (10 μL; 20 μM) in methanol (MeOH)/1% acetic acid (HAc) was added to oxidize the radionuclide. After 30 s, conjugated PL (large/small PL: 100 μL, 1 mg/mL) in 0.2 M acetate buffer, pH 5.5, was added to the reaction vial. The labeling of the polymer proceeded under agitation for 1 min, after which NIS in MeOH/1% HAc (large PL 3.1 μL, 22.2 mM; small PL 2.15 μL, 22.2 mM) was added to the reaction mixture to exchange the remaining tin groups for iodine. After 1 min of agitation, the reaction was quenched with ascorbic acid in water (5 μL, 0.27 M). The polymer product was isolated in PBS using a NAP-10 column with a radiochemical yield of > 80%.

The Radiochemical Yield (RCY) and Radiochemical Purity (RCP) was calculated according to the following formulas:

$$RCY = \frac{{A\,product}}{{A\,tot}}$$

$$RCP = \frac{{APL}}{{A\,product}}$$

Where Aproduct represent the activity of the product fraction after size exclusion purification, Atot the total amount of activity added to the reaction and APL represents the activity of the actual polymer fraction.

#### Quality control

The radiochemical purity was measured on a miniGITA Dual radio TLC scanner with an alpha probe, Elysia-raytest Straubenhardt (Germany) using iTLC-SG glass microfiber chromatography paper impregnated with silica gel, Agilent Technologies, Folsom CA (USA). After a TLC run of the astatinated effector molecule in ethanol, the strip was placed on the radio TLC scanner. In such conditions, free ^211^At migrated to the frontline while the effector molecule stayed at the deposit spot (Timperanza et al. 2023).

### Pretargeting agent synthesis

The pretargeting agent was synthesized by conjugating TCO to the mAb trastuzumab according to our previous study (Timperanza et al. 2023). In brief, the antibody was purified twice from the injection vial stock solution (21 mg/mL), first on a NAP-5 then on a NAP-10 column and eluted in 0.2 M carbonate buffer at pH 8.5 to a concentration of 5 mg/mL. TCO was dissolved in DMF to a concentration of 10 mg/mL. Then, TCO (26.7 μL) was added to 1.5 mL of the mAb solution, rendering an excess of 20:1 (TCO: mAb), and the mixture was left to react for 2.5 h at RT protected from light. When the reaction was complete, the conjugated antibody was purified on a NAP-5 column and the product was eluted in PBS at a concentration of 2.5 mg/mL. According to literature, this will result in the attachment of between 2 and 6 (less than 10) TCO moieties per antibody, enough to ensure success in the in vivo experiments without affecting the antibody’s immunoreactivity (Qiu et al. 2019).

### Stability in serum

Stability studies were carried out using a solution of HSA in PBS at a concentration of 40 mg/mL, similar to the one found in the blood. From 0.1 mg/mL of effectors (10 and 21 kDa), 100 μL of each were transferred to 900 μL of serum solution and incubated at 37 °C. The same protocol was used for the respective clicked products. After 24 h the structural integrity of the polymers and their respective clicked products was evaluated on an FPLC system ÄKTA purifier, Amersham Biosciences, GE Healthcare, (Sweden) with a Superdex 200 10/300 GL column Cytiva-GE Healthcare Bio-Sciences AB, Uppsala (Sweden) using a flow rate of 0.5 mL/min (225–280 nm). The FPLC fractions were collected and measured using a NaI (Tl) detector (Wizard 2480) to check for radiolysis products.

### Biodistribution study of the astatinated effectors

The biodistribution of the two sizes of astatinated effector molecules was evaluated in healthy female normal Balb/c mice 4 weeks of age. Twenty-four mice were divided into two groups (group A 10 kDa; group B 21 kDa), twelve mice for each PL size. The injection solution of both labeled effector molecules was prepared so that the final activity concentration was 7 MBq/mL. Following i.v. injection of the effector molecule (∼ 700 kBq in 100 μL of PBS), a total of 3 mice per group were sacrificed by cervical dislocation after 1, 3, 6, and 24 h. Whole blood was collected by cardiac puncture immediately after the animals were killed, and tissues including salivary glands, throat (including thyroid), lungs, heart, stomach, liver, spleen, small intestine, large intestine, kidneys, intraperitoneal fat, femur, and muscle were dissected. The tissues were accurately weighed, and the activity was measured using a NaI (Tl) detector (Wizard 2480).

### Biodistribution study of the astatinated monoclonal antibody

The biodistribution of the astatinated mAb MX-35 was also evaluated in healthy female normal Balb/c mice. Four mice were injected with solution of labeled MX-35 with an activity concentration of 7 MBq/mL. Following the i.v. injection (∼ 700 kBq in 100 μL of PBS), the 4 mice were sacrificed by cervical dislocation after 24 h. Whole blood was collected by cardiac puncture immediately after the animals were killed, and tissues including salivary glands, throat (including thyroid), lungs, heart, stomach, liver, spleen, small intestine, and kidneys were removed. The tissues were accurately weighed, and the activity was measured using a NaI (Tl) detector (Wizard 2480).

### Cell study

The efficacy of the pretargeting system was evaluated by measuring the binding of the labeled small effector molecule (TzPL; 10 kDa), which showed a better pharmacokinetics profile, to the pretargeting agent (Trastuzumab-TCO) previously loaded onto SKOV-3 cells.

#### Pretargeting agent uptake to cells

The cells were prepared in single-cell suspension at a concentration of 2.5 × 10^6^ cells/mL and divided into triplets of 333 μL each for the evaluation of both PL effector molecule. The amount of the pretargeting molecule to be used (in absolute numbers based on antibody concentration) was based on previous estimations of the number of binding sites for Trastuzumab on SKOV-3 cells (roughly 1.5 × 10^6^ sites/cell) and determined so as to ensure that the antibody-conjugate was in excess (circa 40 times) over available binding sites. Therefore, a constant amount of 12.5 μg of Trastuzumab-TCO was added, and the cells were incubated at room temperature, with gentle agitation, covered from the light for 2.5 h to avoid the isomeration of the TCO to Cis-cyclooctene (CCO), after which, the cells were centrifuged for 5 min at 3000 rpm, washed with PBS, and resuspended in 480 μL of medium.

#### Effector molecule uptake to cells

To ensure that a deficit of effector compared to pretargeting agent was used, a relation of 1:10 of effector compared to assumed bound pretargeting agents was chosen. Thereby, 20.8 μL of the 10 kDa astatinated effector molecule (1 × 10^− 12^ mol/mL) were added in triplets to the cells and incubated at room temperature, under gentle agitation, and protected from light. An aliquot of 166 μL was taken at three different time points to check the binding by measuring the activity left in the cell pellets after centrifuging and washing.

### Electronic supplementary material

Below is the link to the electronic supplementary material.

Supplementary Material 1

## Data Availability

Not applicable.

## Abbreviations

%IA/g: Percentage of Injected Activity per gram
ATE: Activated Tin Ester
DMSO: Dimethyl Sulfoxide
FPLC: Fast Protein Liquid Chromatography
HAc: Acetic Acid
HSA: Human Serum Albumin
LET: Linear Energy Transfer
mAb: Monoclonal Antibody
MeOH: Methanol
NIS: N-Iodo-Succinimide
PL: Poly-L-Lysine
PRIT: Pretargeted RadioImmuno Therapy
RCP: Radiochemical purity
RCY: Radiochemical Yield
RIT: Radioimmunotherapy
RT: Room Temperature
SEM: Standard Error of the Mean
TAT: Targeted Alpha Therapy
TCO: Transcyclooctene
Tz: Tetrazine
TzPL: Tetrazine-substituted Poly-L-Lysine
