# Supplementary material for: In vitro and in vivo evaluation of a tetrazine-conjugated poly-L-lysine effector molecule labeled with astatine-211
Source: EJNMMI Radiopharm Chem. 2024 May 22;9:43. doi: 10.1186/s41181-024-00273-z (PMC11111624; doi:10.1186/s41181-024-00273-z)
Supplement: Supplementary file 1 — Supplementary Material 1 [file 41181_2024_273_MOESM1_ESM.docx]

**SUPPLEMENTARY MATERIAL**

**In vitro and in vivo evaluation of a tetrazine-conjugated poly-L-lysine effector molecule labeled with Astatine-211**

**Chiara Timperanza^1,^ *, Holger Jensen^2^, Ellinor Hansson^1^, Tom Bäck^1^, Sture Lindegren^1^ and Emma Aneheim^1,3^**

^1^ Department of Medical Radiation Sciences, Institute of Clinical Sciences, Sahlgrenska Academy, University of Gothenburg, 413 45 Gothenburg, Sweden;

^2^ Department of Clinical Physiology and Nuclear Medicine, Cyclotron and Radiochemistry unit, Rigshospitalet, Blegdamsvej 9, 2100 Copenhagen, Denmark;

^3^ Department of Oncology, Sahlgrenska University Hospital, Region Västra Götaland, 413 45 Gothenburg, Sweden;

^*^Correspondence: [chiara.timperanza@gu.se](mailto:chiara.timperanza@gu.se)

Email addresses:

[chiara.timperanza@gu.se](mailto:chiara.timperanza@gu.se)

[Holger.Jan.Jensen@regionh.dk](mailto:Holger.Jan.Jensen@regionh.dk)

[ellinor.hansson@gu.se](mailto:ellinor.hansson@gu.se)

[tom.back@radfys.gu.se](mailto:tom.back@radfys.gu.se)

[sture.lindegren@radfys.gu.se](mailto:sture.lindegren@radfys.gu.se)

[emma.aneheim@radfys.gu.se](mailto:emma.aneheim@radfys.gu.se)

a)

b)

d)

c)

**Figure S1:** Radioactivity distribution after fast protein liquid chromatography (FPLC) fraction collection of the radiolabeled effector molecules (10 and 21 kDa) and their respective click products. Panel (a) shows astatine-labeled 10 kDa polymer after incubation in human serum albumin (HSA)/PBS solution for 24 hours at 37°C. Panel (b) shows the click product (from 10 kDa PL and Trastuzumab-TCO) before and after incubation in HSA/PBS solution for 24 hours at 37°C. Panel (c) shows astatine-labeled 21 kDa polymer after incubation in HSA/PBS. Panel (d) shows the click product (from 21 kDa PL and Trastuzumab-TCO) before and after incubation in HSA/PBS for 24 hours at 37°C.
